# Statement of Retraction: A regulatory circuit of circ-MTO1/miR-17/QKI-5 inhibits the proliferation of lung adenocarcinoma

**DOI:** 10.1080/15384047.2023.2240179

**Published:** 2023-09-03

**Article title**: A regulatory circuit of circ-MTO1/miR-17/QKI-5 inhibits the proliferation of lung adenocarcinoma

**Authors**: Binbin Zhang, Maolin Chen, Nan Jiang, Kefeng Shi, Runlin Qian

**Journal**: *Cancer Biology & Therapy*

**DOI**: 10.1080/15384047.2019.1598762

The Publisher was contacted regarding concerns about the duplication of Figures 2C and 2H from articles that have been previously published in other articles with no connection to the above-mentioned manuscript.

When approached for an explanation, the authors were unable to provide their original data or any necessary supporting information. The authors agree with the decision to retract the article.

Our decision-making was informed by our policy on publishing ethics and integrity and the COPE guidelines on retraction.

The retracted article will remain online to maintain the scholarly record, but it will be digitally watermarked on each page as “Retracted”.

